# Supplementary material for: DNA methyltransferase 3A controls intestinal epithelial barrier function and regeneration in the colon
Source: Nat Commun. 2022 Oct 21;13:6266. doi: 10.1038/s41467-022-33844-2 (PMC9587301; doi:10.1038/s41467-022-33844-2)
Supplement: Supplementary file 1 — Supplementary information [file 41467_2022_33844_MOESM1_ESM.pdf]

Supplementary Information

DNA methyltransferase 3A controls intestinal epithelial barrier function and regeneration in the colon

Fazio et al.

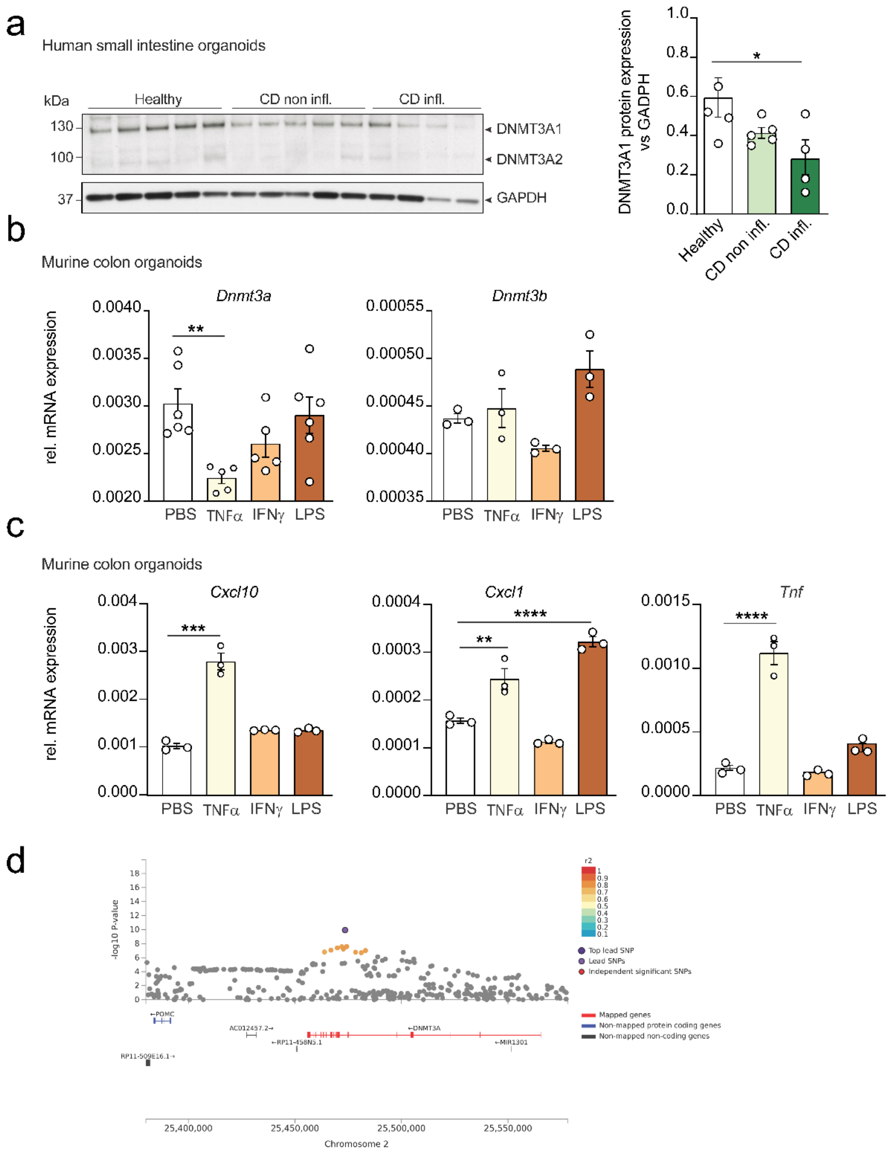

Supplementary Figure 1: DNMT3A expression is dysregulated during inflammation. (**a**) Immunoblot and quantification of DNMT3A protein expression in human small intestinal organoid lysates derived from healthy (n=5), CD non inflamed (non-inf) (n=5) and CD (inflamed) infl (n=4) tissues. (**b**) Gene expression analysis of Dnmt3a (n=6) and Dnmt3b (n=3) and (**c**) Cxcl1 and Tnfa in murine organoids stimulated for 24h with TNFa (50ng/ml), IFNg (50ng/ml), LPS (50ng/ml) and PBS as control (n=3). Gapdh was used as housekeeping gene. The values represent mean ± SEM of two independent experiments. Statistical analysis was performed using one-way ANOVA together with Tukey post hoc test. (**d**) GWAS regional association plots for the known CD risk locus 2p23.3 14,15 which comprises two independent genome-wide significant association signals (loci) for CD; intronic lead signal #1 at ADCY3 and another intronic lead signal #2 at DNMT3A). Weaker non-genome-wide significant association signals are also present at the same locus for UC. Shown are the -log10 P-values (two-sided) from CD GWAS summary statistics with regard to the physical location of SNP markers. Purple filled circle: lead SNP; Other filled circles: analyzed SNPs where the fill color corresponds to the strength of linkage disequilibrium (r2) with the lead SNP (see legend in the upper right corner for color coding); positions and gene annotations correspond to NCBI human genome build 37 (hg19). Potential candidate genes in red were mapped using the FUMA software 75 based on positional mapping, eQTL mapping, or chromatin interaction mapping, otherwise genes are considered unmapped. *p<0.05, **p<0.01, ***p<0.001, ****p<0.0001

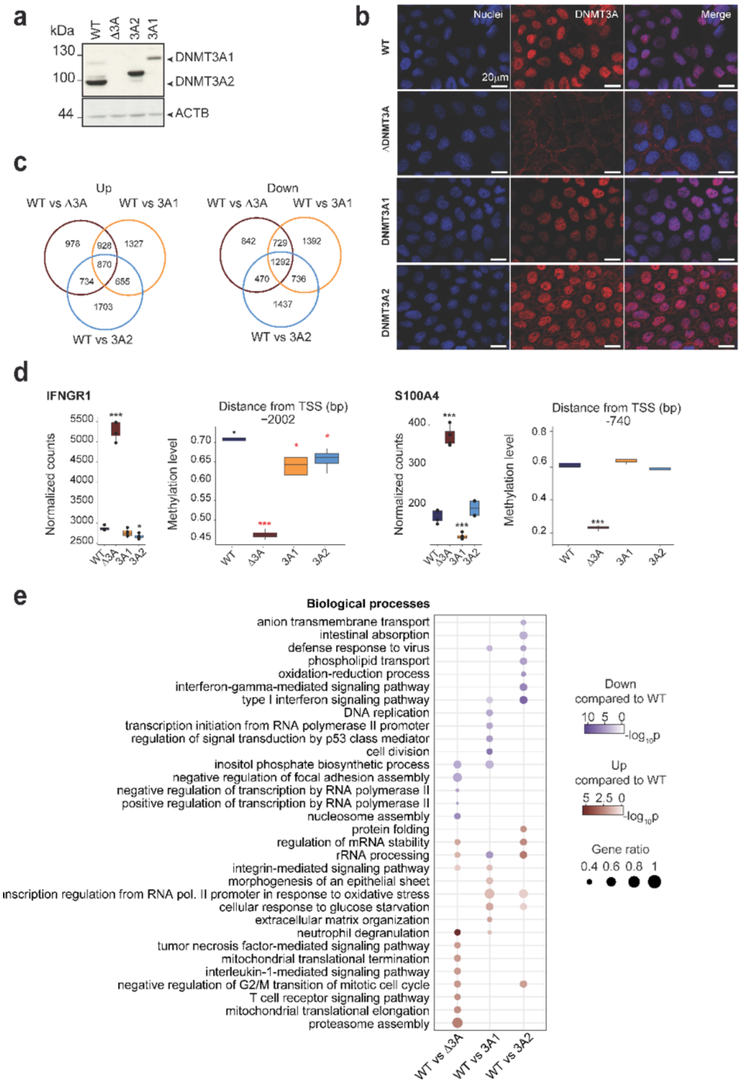

Supplementary Figure 2: Genomic deletion of DNMT3A in Caco-2 cells and subsequent re-expression of individual isoforms. (**a**) Immunoblot analysis of DNMT3A protein expression in WT, ΔDNMT3A, DNMT3A1 and DNMT3A2 Caco-2 cells. Human beta-actin was used as loading control (representative immunoblot from all cell lines used in the study). (**b**) Immunofluorescence staining of DNMT3A in WT, ΔDNMT3A, DNMT3A1 and DNMT3A2 Caco-2 cells (representative IF images from all cell lines used in the study). (**c**) Venn diagram showing numbers of significantly up-regulated and down-regulated transcripts in ΔDNMT3A, DNMT3A1, DNMT3A2, vs. WT (n = 4). (**d**) Summary plot of selected genes displaying DNA methylation and gene expression of IFNGR1 and S100A4 following canonical DNA methylation/gene expression correlation. Left panel indicates gene expression plotted as normalized reads count in each group. Right panels show DNA methylation levels across distance from TSS (n=4). Within each boxplot, the horizontal lines denote the median values and the boxes extend from the 25th to the 75th quartile of the distribution. The vertical lines extend to the most extreme values within 1.5 interquartile range of the 25th and 75th percentile of the distribution. P-values are calculated using the DESeq2 method for the transcriptomic data and the hierarchical linear model (limma) for the methylation data. (**e**) Gene ontology analysis for biological processes of DEGs in ΔDNMT3A, DNMT3A1, DNMT3A2, vs. WT. Dot size is proportional to the gene ratio and colour corresponds to the p-value of enrichment (two-sided). Top selected GO terms are shown. *p<0.05, **p<0.01, ***p<0.001, ****p<0.0001

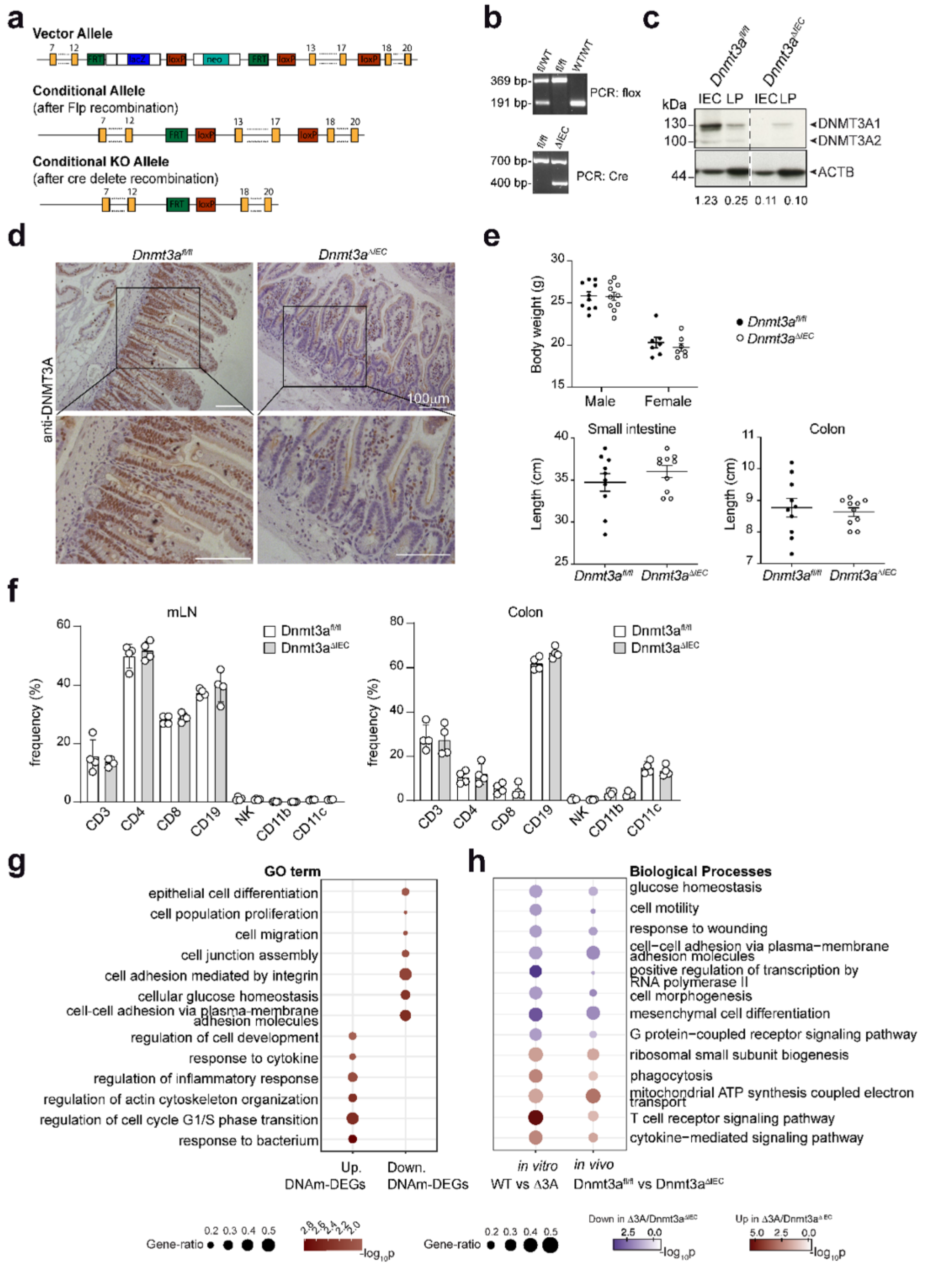

Supplementary Figure 3: Generation of conditional Dnmt3aΔIEC mice. (**a**) Targeting strategy for the generation of conditional Dnmt3a knockout allele. (**b**) Flox and Cre PCRs used for genotyping analysis (representative genotype blot from all animals used in the study). (**c**) Immunoblot analysis of DNMT3A in IECs and LP fractions isolated from colonic tissue. Murine beta-actin was used as loading control (representative immunoblot from all animals used in the study). (**d**) Immunohistochemical staining of Dnmt3a protein in small intestinal tissue from Dnmt3aΔIEC and Dnmt3afl/fl mice. (**e**) Body weight, small intestine and colon length of Dnmt3aΔIEC and Dnmt3afl/fl mice in steady state condition (female n=7, male n=10). (**f**) Flow cytometry analysis of immune cells in mesenteric lymph nodes and colonic lamina propria isolated from Dnmt3afl/fl and Dnmt3a ΔIEC in steady state condition (n=4 biological replicates). The values represent mean ± SEM. (**g, h**) GO terms enriched in up- and down-regulated DNAm-DEGs (DEGs with cis-linked DNA methylation changes) in Dnmt3aΔIEC compared to Dnmt3afl/fl mice (**g**) and Common up- and down-regulated GO terms from in vitro and in vivo DEGs (**h**). Dot size is proportional to the gene ratio and colour corresponds to the p-value of enrichment (two-sided). Top selected GO terms are shown.

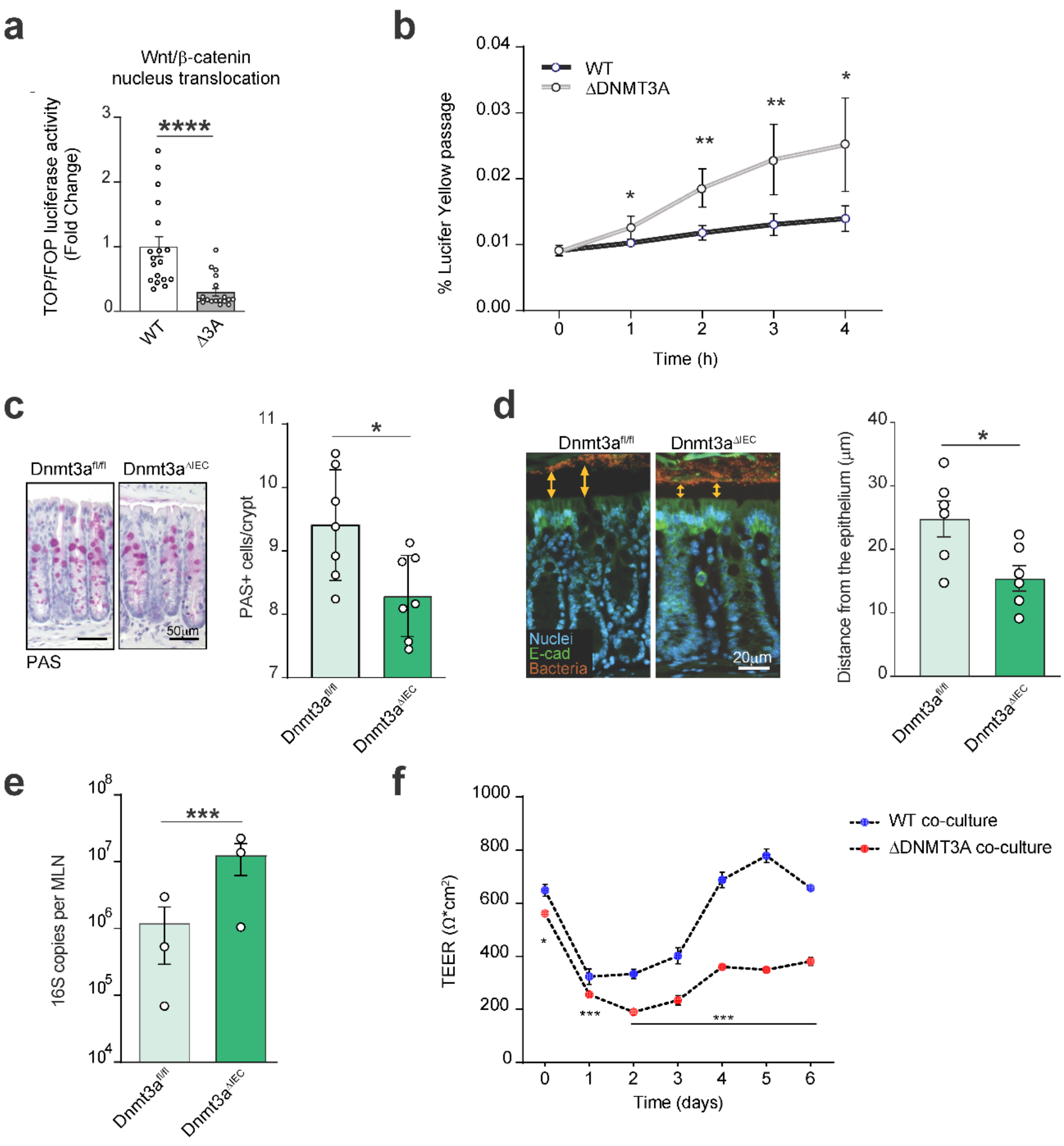

Supplementary figure 4: Loss of Dnmt3a impairs barrier properties at steady state. (**a**) TOP/FOP luciferase reporter assay of Caco-2 WT and ΔDNMT3A (n=18 over 3 independent experiments). (**b**) Percentage of lucifer yellow passage in differentiated ΔDNMT3A and WT Caco-2 monolayers at different time points (n=8 over two independent experiments). (**c**) Representative images and quantification of PAS positive cells from colonic tissue sections of steady state Dnmt3aΔIEC and Dnmt3afl/fl mice (n=7). A minimum of 100 crypts were analyzed per group. Each dot represents each animal. (**d**) Representative images of fluorescent in situ hybridization (FISH) and measurements of distance between intestinal epithelium and luminal content of steady state Dnmt3aΔIEC and Dnmt3afl/fl mice (n=6). Colonic sections were stained for general bacteria 16S (probe EUB338) (dark orange), E-cadherin (green) and nuclei (light blue). Each dot represents each animal. (**e**) Total bacterial DNA amount in mesenteric lymph nodes of steady state Dnmt3aΔIEC and Dnmt3afl/fl mice (n=3). (**f**) TEER measurements of differentiated ΔDNMT3A and WT Caco-2 monolayers co-cultured with activated THP-1 cells (n=3 over 3 independent experiments). The values represent mean ± SEM. Statistical analysis was performed using two-tailed T-test with Mann-Whitney correction. *p<0.05, **p<0.01, ***p<0.001, ****p<0.0001

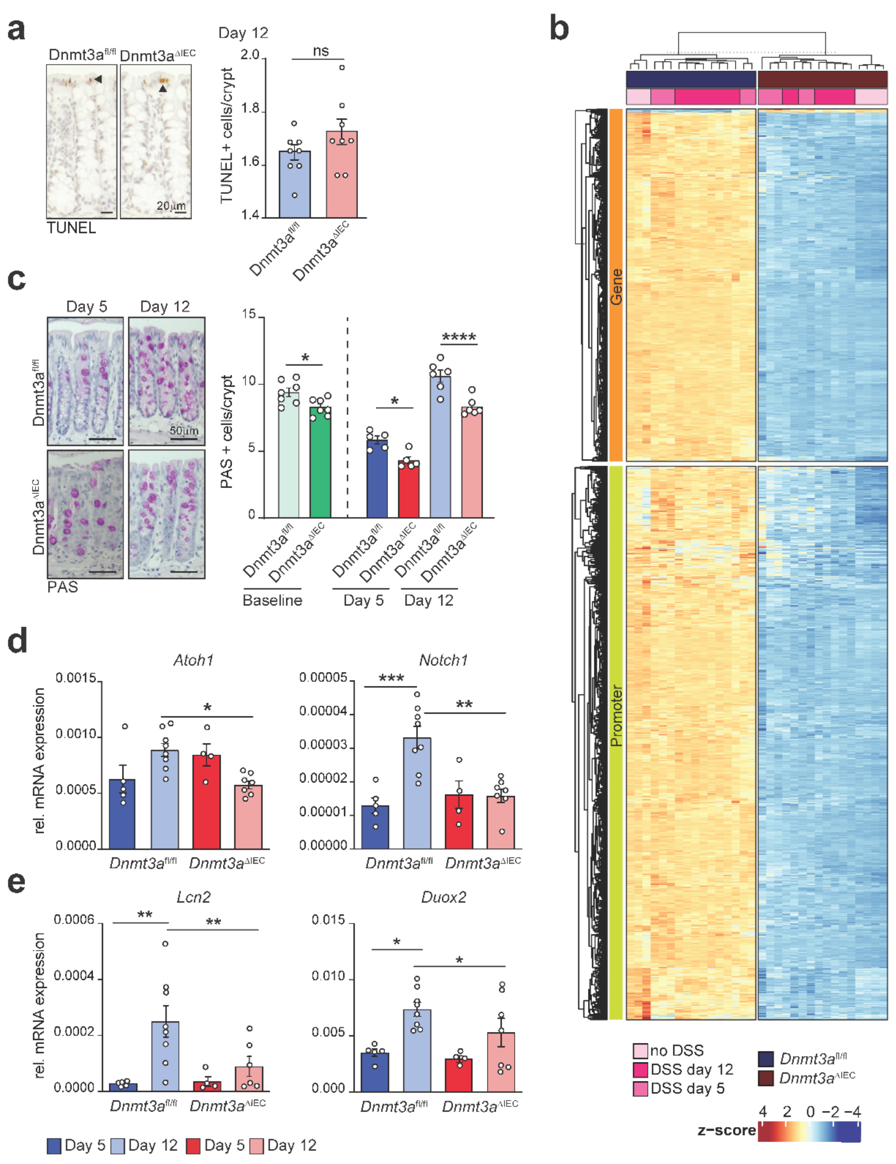

Supplementary figure 5: Dnmt3a is crucial for antimicrobial response after acute inflammation. (**a**) Representative images and quantification of TUNEL stained colonic tissue of Dnmt3aΔIEC and Dnmt3afl/fl mice at day 12 (recovery phase). A minimum of 50 crypts were analyzed per group. Each dot represents each animal (n=8). Arrow heads indicate TUNEL positive cells. (**b**) Heatmaps showing DMRs identified in Dnmt3aΔIEC versus Dnmt3afl/fl IECs at baseline (no DSS; n=4), DSS day5 (n=5) and DSS day12 (n=8). (**c**) Representative images and quantification of PAS positive cells in colonic sections from Dnmt3aΔIEC and Dnmt3afl/fl mice during early inflammation (day 5, n=5), recovery phase (day 12, n=8) and at baseline (shown in Suppl. Fig. 4c). A minimum of 100 crypts were analyzed per group. Each dot represents each animal. (**d**) Relative mRNA expression levels of Atoh1 and Notch1 and (**e**) antimicrobial inflammatory epithelial genes Lcn2 and Duox2 in Dnmt3aΔIEC and Dnmt3afl/fl mice during early inflammation (day 5, n=5) and recovery phase (day 12, n=7/8). Murine beta-actin was used as housekeeping gene. The values represent mean ± SEM. Statistical analysis was performed using two-tailed T-test with Mann-Whitney correction (**a**), one-way ANOVA together with Tukey post hoc test (**d-e**) and one-way ANOVA together with Bonferroni test (**c**). ns= not significant, *p<0.05, **p<0.01, ***p<0.001, ****p<0.0001

Supplementary table 1: Demographic information of enrolled participants

Group	count	male	female	median age	min age	max age
overall	150	73	77	40	18	73
CDinfl	30	16	14	29,5	18	46
CDn-infl	30	13	17	40	18	69
CD	*60*	*29*	*31*	*36*	*18*	*69*
UCinfl	30	15	15	31,5	19	61
UCn-infl	30	15	15	39	18	70
UC	*60*	*30*	*30*	*36,5*	*18*	*70*
HN	30	14	16	54	26	73

Median age: 40
Mean age: 41.87
Age range: 18-73
Treatment: heterogenous group, different treatments, reflecting the clinical scenario

Supplementary Table 2: Clinical characteristics for all participants enrolled for organoid generation

A	B	C	D	E	F	G	H	I	L	M
**1**	N/A	N/A	ileum	healthy	N/A	N/A	N/A	N/A	x	
**2**	N/A	N/A	ileum	healthy	N/A	N/A	N/A	N/A	x	
**3**	N/A	N/A	ileum	healthy	N/A	N/A	N/A	N/A	x	
**4**	N/A	N/A	ileum	healthy	N/A	N/A	N/A	N/A	x	
**5**	N/A	N/A	ileum	healthy	N/A	N/A	N/A	N/A	x	
**6**	20-30	female	ileum	non-inflamed	adalimumab	no	no	no	x	
**7**	40-50	female	ileum	non-inflamed	vedolizumab	no	no	no	x	
**8**	40-50	female	ileum	non-inflamed	vedolizumab	no	no	no	x	
**9**	20-30	male	ileum	non-inflamed	vedolizumab	no	no	sulfasalazine	x	
**10**	20-30	male	ileum	non-inflamed	infliximab	no	no	no	x	
**11**	40-50	male	ileum	mild	infliximab	no	no	no	x	
**12**	40-50	male	ileum	mild	infliximab	no	no	no	x	
**13**	30-40	male	ileum	mild	vedolizumab	no	no	mesalamine	x	
**14**	40-50	male	ileum	moderate	-	no	no	no	x	
**15**	N/A	N/A	sigma	healthy	N/A	N/A	N/A	N/A	x	
**16**	N/A	N/A	sigma	healthy	N/A	N/A	N/A	N/A	x	
**17**	N/A	N/A	sigma	healthy	N/A	N/A	N/A	N/A	x	
**18**	N/A	N/A	sigma	healthy	N/A	N/A	N/A	N/A	x	x
**19**	40-50	male	sigma	non-inflamed	-	0	no	no	x	
**20**	20-30	male	sigma	non-inflamed	infliximab	0	no	no	x	
**21**	50-60	female	sigma	non-inflamed	-	budesonide	no	no	x	
**22**	20-30	female	sigma	non-inflamed	-	budesonide	no	no	x	
**23**	40-50	male	sigma	non-inflamed	-	no	no	mesalamine	x	x
**24**	20-30	male	sigma	moderate	-	no	no	mesalamine	x	
**25**	40-50	male	sigma	moderate	infliximab	no	no	no	x	
**26**	20-30	female	sigma	moderate	adalimumab	no	no	no	x	x
**27**	20-30	male	sigma	mild	infliximab	no	no	no	x	
**28**	20-30	male	sigma	severe	adalimumab	no	no	no	x	
**29**	20-30	female	sigma	N/A	N/A	N/A	N/A	N/A	x	

A Organoid ID
B range age
C sex
D region of sampling
E inflammation grade
F biological treatment
G concomittant use of corticosteroids
H concomitant immunomodulator use
I concomitant 5-ASA use
L used for immunoblot analysis
M used for gene expression analysis

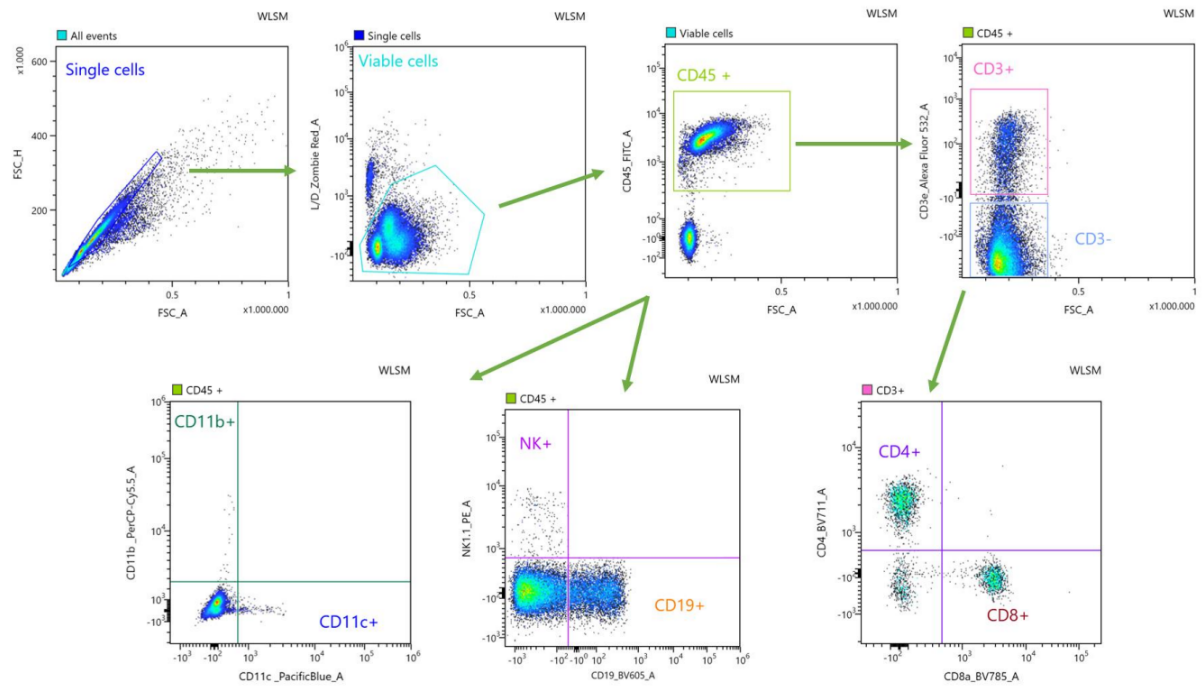

Gating strategy related to Suppl. Figure 3f: Gating was performed excluding doublets and debris using FSC_H and FSC_A events distribution. Singlets were tested for viable population (Zombie Red negative) using Zombie Red'M Fixable Viability Kit Biolegend. Viable cells were plotted using CD45 as leukocyte marker. Positive CD45 events were tested for CD3 expression and CD3 positive events were plotted using CD4 and CD8 as T-cell markers for Cytotoxic and Helper T-cells. CD11b, CD11c, NK1.1 and CD19 were used for Monocytes, Dendritic cells, Natural Killer cells and B cells respectively.
