# Supplementary material for: DNA methyltransferase 3A controls intestinal epithelial barrier function and regeneration in the colon
Source: Nat Commun. 2022 Oct 21;13:6266. doi: 10.1038/s41467-022-33844-2 (PMC9587301; doi:10.1038/s41467-022-33844-2)
Supplement: Supplementary file 3 — Description of Additional Supplementary Files [file 41467_2022_33844_MOESM3_ESM.pdf]

Description of Additional Supplementary Files

File Name: Supplementary Data 1
Description: CD TWAS association results for 44 imputable genes from the established IBD risk locus 2p23.3 including DNMT3A (extended region chr2:24,999kb-27,900kb to capture long range cis eQTL effects). UC TWAS association results for 44 imputable genes from the established IBD risk locus 2p23.3 including DNMT3A.

File Name: Supplementary Data 2
Description: List of significantly differentially expressed genes between WT and Δ3A, 3A1 and 3A2 Caco-2 cells.

File Name: Supplementary Data 3
Description: List of GO terms enriched in DEGs from in vitro experiments.

File Name: Supplementary Data 4
Description: List of differentially methylated regions (DMRs) in ΔDNMT3A Caco-2 cells compared to WT Caco-2 cells.

File Name: Supplementary Data 5
Description: Comparison of transcription-DNA methylation correlation between *in vitro* and *in vivo*.

File Name: Supplementary Data 6
Description: List of GO terms enriched in in vitro rescued genes.

File Name: Supplementary Data 7
Description: List of differentially methylated regions (DMRs) in Dnmt3a$^{\triangle IEC}$ compared to Dnmt3a$^{fl/fl}$ mice.

File Name: Supplementary Data 8
Description: List of significantly differentially expressed genes between Dnmt3a$^{fl/fl}$ and Dnmt3a$^{\Delta IEC}$ mice.

File Name: Supplementary Data 9
Description: List of GO terms enriched in in vivo DEGs.

File Name: Supplementary Data 10
Description: List of GO terms enriched in in vivo DNAm-linked DEGs.

File Name: Supplementary Data 11
Description: List of GO terms enriched in DEGs shared between *in vitro* and *in vivo.*

File Name: Supplementary Data 12
Description: Comparison of log fold change of in vitro and in vivo DEGs.

File Name: Supplementary Data 13
Description: List of GO terms enriched in DMPs at day 5 and day 12 of DSS treatment in $Dnmt3a^{fl/fl}$ and $Dnmt3a^{\triangle IEC}$ mice.
