# Supplementary material for: DNA methyltransferase 3A controls intestinal epithelial barrier function and regeneration in the colon
Source: Nat Commun. 2022 Oct 21;13:6266. doi: 10.1038/s41467-022-33844-2 (PMC9587301; doi:10.1038/s41467-022-33844-2)
Supplement: Supplementary file 17 — Reporting Summary [file 41467_2022_33844_MOESM17_ESM.pdf]

nature portfolio

Corresponding author(s): Philip Rosenstiel
Last updated by author(s): Sep 20, 2022

# Reporting Summary

Nature Portfolio wishes to improve the reproducibility of the work that we publish. This form provides structure for consistency and transparency in reporting. For further information on Nature Portfolio policies, see our Editorial Policies and the Editorial Policy Checklist.

## Statistics

For all statistical analyses, confirm that the following items are present in the figure legend, table legend, main text, or Methods section.

| n/a | Confirmed | |
|---|---|---|
| ☐ | ☒ | The exact sample size (*n*) for each experimental group/condition, given as a discrete number and unit of measurement |
| ☐ | ☒ | A statement on whether measurements were taken from distinct samples or whether the same sample was measured repeatedly |
| ☐ | ☒ | The statistical test(s) used AND whether they are one- or two-sided<br>*Only common tests should be described solely by name; describe more complex techniques in the Methods section.* |
| ☒ | ☐ | A description of all covariates tested |
| ☐ | ☒ | A description of any assumptions or corrections, such as tests of normality and adjustment for multiple comparisons |
| ☐ | ☒ | A full description of the statistical parameters including central tendency (e.g. means) or other basic estimates (e.g. regression coefficient) AND variation (e.g. standard deviation) or associated estimates of uncertainty (e.g. confidence intervals) |
| ☐ | ☒ | For null hypothesis testing, the test statistic (e.g. *F*, *t*, *r*) with confidence intervals, effect sizes, degrees of freedom and *P* value noted<br>*Give P values as exact values whenever suitable.* |
| ☒ | ☐ | For Bayesian analysis, information on the choice of priors and Markov chain Monte Carlo settings |
| ☐ | ☒ | For hierarchical and complex designs, identification of the appropriate level for tests and full reporting of outcomes |
| ☐ | ☒ | Estimates of effect sizes (e.g. Cohen's *d*, Pearson's *r*), indicating how they were calculated |

*Our web collection on statistics for biologists contains articles on many of the points above.*

## Software and code

Policy information about availability of computer code

| | |
|---|---|
| Data collection | Sequencing Base calling: bcl2fastq v2.20.0.422 |
| Data analysis | RNA-seq data analysis:<br>Trim Galore 0.4.4, STAR 2.5.2b, featureCounts 1.5.2, DESeq2 1.20.0, topGO 2.32.0<br>DNA methylation analysis:<br>RnBeads 1.12.1, topGO 2.32.0<br>Statistical analysis:<br>GraphPad Prism 9<br>Microscopy analysis:<br>ZEN 3.4 Blue edition<br>AxioVision Imaging software<br>ImageJ 1.53e<br>Flow cytometry analysis:<br>SA3800 software Spectral Analyzer |

For manuscripts utilizing custom algorithms or software that are central to the research but not yet described in published literature, software must be made available to editors and reviewers. We strongly encourage code deposition in a community repository (e.g. GitHub). See the Nature Portfolio guidelines for submitting code & software for further information.

## Data

Policy information about availability of data

All manuscripts must include a data availability statement. This statement should provide the following information, where applicable:

- Accession codes, unique identifiers, or web links for publicly available datasets
- A description of any restrictions on data availability
- For clinical datasets or third party data, please ensure that the statement adheres to our policy

RNA-sequencing data of experiments using Caco-2 cells and mouse model (GEO database, accession number GSE210714, https://www.ncbi.nlm.nih.gov/geo/query/acc.cgi?acc=GSE210714)
DNA methylation profiling data from Caco-2 cells and mouse model (GEO database, accession number GSE210721, https://www.ncbi.nlm.nih.gov/geo/query/acc.cgi?acc=GSE210721)
The analysis output files are provided as supplementary material.

## Human research participants

Policy information about studies involving human research participants and Sex and Gender in Research.

| | |
|---|---|
| Reporting on sex and gender | We have reported sex/gender on all human subjects involved in Supplementary Table 1 and Table 2. |
| Population characteristics | The clinical characteristics of the UC, CD and HC patients involved in the gene expression analysis of figure 1a were provided in Supplementary Table 1. The clinical characteristics of the CD and HC for gene expression and protein analysis of figure 1b, 1c and Supplementary figure 1a were provided in Supplementary Table 2. The clinical characteristics of the UC and CD patients in supplementary figure 1d were provided in supplementary table 3a and 3b. |
| Recruitment | Patients in this study were recruited from the University Hospital Schleswig-Holstein, Kiel, Germany. All patients and healthy individuals information and samples were collected based on clinical requirement for diagnosis. Healthy individuals were recruited from the same clinical set-up, where they presented for clinical workup of nonspecific gastrointestinal symptoms or colorectal cancer screening, respectively. Individuals were considered healthy if they showed no signs of pathology by colonoscopy, histopathology of colonic routine biopsies as well as no other major diseases during diagnostic workup. There were no self-selection bias or other biases. |
| Ethics oversight | All patients and healthy individuals gave informed consents for collection of tissue collection. All procedures were performed in accordance with institutional guidelines and were approved by the ethics committee of the medical faculty of Kiel University before the study (B231/98) and (B231/13). |

Note that full information on the approval of the study protocol must also be provided in the manuscript.

# Field-specific reporting

Please select the one below that is the best fit for your research. If you are not sure, read the appropriate sections before making your selection.

☒ Life sciences ☐ Behavioural & social sciences ☐ Ecological, evolutionary & environmental sciences

For a reference copy of the document with all sections, see nature.com/documents/nr-reporting-summary-flat.pdf

# Life sciences study design

All studies must disclose on these points even when the disclosure is negative.

| | |
|---|---|
| Sample size | No statistical methods were used to predetermine sample sizes. Variable sample sizes were chosen depending on the addressed questions and based on published literature. Samples size for all experiments are described in each figure description. |
| Data exclusions | No data was excluded from the analysis. |
| Replication | All experiments (except for the individual in vivo experiments, which are only allowed once with an adequate number of animals) were conducted at least two times independently. Each replication was successful. |
| Randomization | Samples were randomly allocated into the study. |
| Blinding | All microscopy picture quantifications (PAS, TUNEL, FISH, TEM) were conducted by blinded investigators. For the other experiments, data collection and analysis could not be influenced by the investigators, therefore blinding was not applied. |

# Reporting for specific materials, systems and methods

We require information from authors about some types of materials, experimental systems and methods used in many studies. Here, indicate whether each material, system or method listed is relevant to your study. If you are not sure if a list item applies to your research, read the appropriate section before selecting a response.

## Materials & experimental systems

| n/a | Involved in the study |
|---|---|
| ☐ | ☒ Antibodies |
| ☐ | ☒ Eukaryotic cell lines |
| ☒ | ☐ Palaeontology and archaeology |
| ☐ | ☒ Animals and other organisms |
| ☒ | ☐ Clinical data |
| ☒ | ☐ Dual use research of concern |

## Methods

| n/a | Involved in the study |
|---|---|
| ☒ | ☐ ChIP-seq |
| ☐ | ☒ Flow cytometry |
| ☒ | ☐ MRI-based neuroimaging |

## Antibodies

Antibodies used

FACS Antibody 1) Fc-Block (CD16/CD32). Anti ms CD16/CD32 _isotype: Rat IgG2b,k_nr: 553142
FACS Antibody 2) CD45-FITC (BioLegend) 1:100. Anti ms. Color: FITC_isotype: Rat igG2b_nr: 1034107
FACS Antibody 3) NK1.1-PE (eBioscience) 1:80. Anti ms. Color: PE_isotype: Mouse IgG2a, κPE_nr: 12-5941-81
FACS Antibody 4) CD3e-AF532 (eBioscience). Anti ms. Color:Alexa Fluor 532_isotype: Rat IgG2b, k_nr: 58-0032-82
FACS Antibody 5) CD11b-PerCP/Cy5.5 (Biolegend) 1:50. Anti ms. Color: PerCP 5,5_isotype:Rat igg2b_nr:101228
FACS Antibody 6) CD4-BV711 (BioLegend) 1:200. Anti ms. Color: Brilliant Violet 711_isotype: Rat igG2b_nr: 100447
FACS Antibody 7) CD19-BV605 (BioLegend) 1:100. Anti ms. Color: Brilliant Violet 605__isotype:Rat igg2a_nr:115539
FACS Antibody 8) CD8-BV785 (BioLegend) 1:100. Anti ms_Color Brilliant Violet 785_ isotype: Rat IgG2aκ_nr: 100749
FACS Antibody 9) CD11c-PacificBlue(BioLegend)1:50.Anti ms_Color:Pacific Blue_isotype: Armenian hamster igG_nr:117321
L/D stain: Zombie Red™ Fixable Viability Kit Biolegend_nr:423109
WB Antibody 1) DNMT3A: Monoclonal Mouse IgG2B Clone # 681615, R&D
WB Antibody 2) GAPDH: Monoclonal Mouse IgG1 κ GAPDH antibody, clone #0411; #sc-47724 Santa Cruz Biotechnology
WB antibody 3) ACTB: ,Monoclonal Mouse IgG1 κ, clone #C-2, #sc-8432, Santa Cruz Biotechnology
FISH antibody 1) Anti-E Cadherin, 610182: Mouse IgG2a, κ clone #36-E BD biosciences
IF antibody 1) ZO-1 Polyclonal Antibody, Rabbit / IgG 40-2200 Invitrogen (Life Technologies)
Secondary antibody: 1) Goat / IgG polyclonal anti mouse Alexa Fluor™ Plus 488-conjugated
2) Goat anti-mouse immunoglobulin G, Jackson ImmunoResearch

Validation

All antibodies were validated on relevant cells/tissues/lysates before use in the study. Own results were compared to results from the respective vendors.

FACS Antibody 1) Data sheet from website: https://www.bdbiosciences.com/content/bdb/paths/generate-tds-document.us.553141.pdf. Citations and references can be found in the following page: https://www.bdbiosciences.com/en-us/products/reagents/flow-cytometry-reagents/research-reagents/single-color-antibodies-ruo/purified-rat-anti-mouse-cd16-cd32-mouse-bd-fc-block.553141.

FACS Antibody 2) Data sheet from website: https://www.biolegend.com/en-us/products/fitc-anti-mouse-cd45-antibody-99?pdf=true&displayInline=true&leftRightMargin=15&topBottomMargin=15&filename=FITC%20anti-mouse%20CD45%20Antibody.pdf. Additional publications: Podd BS, et al. 2006. J. Immunol. 176:6532. (FC, CMCD) PubMed. Haynes NM, et al. 2007. J. Immunol. 179:5099. (FC). Ledbetter JA, et al. 1979. Immunol. Rev. 47:63. (IP).

FACS Antibody 3) Data sheet from website: https://www.thermofisher.com/order/genome-database/dataSheetPdf?producttype=antibody&productsubtype=antibody_primary&productId=12-5941-81&version=243.
Additional publications: 113 references (reported in the manufacturer´s website above).

FACS Antibody 4) All information can be found at the manufacturer´s website: https://www.fishersci.de/shop/products/cd3-mouse-anti-human-alexa-fluor-532-clone-ucht1-ebioscience-2/p-7091560.

FACS Antibody 5) Data sheet from website: https://www.biolegend.com/en-us/products/percp-cyanine5-5-anti-mouse-human-cd11b-antibody-4257?pdf=true&displayInline=true&leftRightMargin=15&topBottomMargin=15&filename=PerCP/Cyanine5.5%20anti-mouse/human%20CD11b%20Antibody.pdf. https://www.biolegend.com/en-us/products/percp-cyanine5-5-anti-mouse-human-cd11b-antibody-4257?GroupID=BLG10552. 33 Application references and 136 citations are available at the above mentioned website.

FACS Antibody 6) Data sheet from website: https://www.biolegend.com/de-de/products/brilliant-violet-711-anti-mouse-cd4-antibody-10706?pdf=true&displayInline=true&leftRightMargin=15&topBottomMargin=15&filename=Brilliant%20Violet%20711%E2%84%A2%20anti-mouse%20CD4%20Antibody.pdf. Product information: https://www.biolegend.com/de-de/products/brilliant-violet-711-anti-mouse-cd4-antibody-10706?GroupID=BLG4211.13 Application references and 16 Product citations are available at the above mentioned website

FACS Antibody 7) Data sheet from website: https://www.biolegend.com/en-us/productstab/brilliant-violet-605-anti-mouse-cd19-antibody-7645?pdf=true&displayInline=true&leftRightMargin=15&topBottomMargin=15&filename=Brilliant%20Violet%20605%E2%84%A2%20anti-mouse%20CD19%20Antibody.pdf. Product information: https://www.biolegend.com/en-us/productstab/brilliant-violet-605-anti-mouse-cd19-antibody-7645?GroupID=BLG10556. 13 Application references and 28 Product citations are available at the above mentioned website.

FACS Antibody 8) Data sheet from website: https://www.biolegend.com/en-us/neuroscience-1/brilliant-violet-785-anti-mouse-cd8a-antibody-7957?pdf=true&displayInline=true&leftRightMargin=15&topBottomMargin=15&filename=Brilliant%20Violet%20785%E2%84%A2%20anti-mouse%20CD8a%20Antibody.pdf. https://www.biolegend.com/en-us/neuroscience-1/brilliant-violet-785-anti-mouse-cd8a-antibody-7957?GroupID=BLG6765. 30 Application references and 46 Product citations are available at the above mentioned website.

FACS Antibody 9) Data sheet from website https://www.biolegend.com/en-us/products/pacific-blue-anti-mouse-cd11c-antibody-3864?pdf=true&displayInline=true&leftRightMargin=15&topBottomMargin=15&filename=Pacific%20Blue%E2%84%A2%20anti-mouse%20CD11c%20Antibody.pdf. Additional information can be found at the following link: https://www.biolegend.com/en-us/products/pacific-blue-anti-mouse-cd11c-antibody-3864?GroupID=BLG11937. 24 Application references and 31 Product citations can be found at the above mentioned link.
L/D stain: self validated using cells trated with staurosporin (cell death inducer). Product information and data sheet can be found at the following website: https://www.biolegend.com/en-us/products/zombie-red-fixable-viability-kit-9338?GroupID=BLG2181.

WB antibody 1) DNMT3A: information on this product can be found at producer website https://www.rndsystems.com/products/human-mouse-dnmt3a-antibody-681615_mab63151

WB antibody 2) GAPDH: information on this product can be found at producer website . https://www.scbt.com/p/gapdh-antibody-0411/. This antibody was cited in 2677 scientific publications.

WB antibody 3) ACTB: Data sheet from website https://datasheets.scbt.com/sc-8432.pdf. Further details of this antibody can be found at https://www.scbt.com/p/actin-antibody-c-2/. This antibody was cited in 2307 scientific publications.

FISH antibody 1) E-Cad. Data sheet from webstite: Purified Mouse Anti-E-Cadherin (bdbiosciences.com). This product was cited in 5 development references.

IF antibody 1) ZO-1. Data sheet from the website: ZO-1 Antibody (40-2200) (thermofisher.com). 76 publications cited this product.

# Eukaryotic cell lines

Policy information about cell lines and Sex and Gender in Research

| | |
|---|---|
| Cell line source(s) | Caco-2 cell line were purchased from German Collection of Microorganisms and Cell Cultures (DSMZ).<br>THP-1 cell line were purchased from German Collection of Microorganisms and Cell Cultures (DSMZ).<br>HEK-Blue-TLR4 reporter cell line were purchased from Invivogen. |
| Authentication | non of the cell line used were authenticated. |
| Mycoplasma contamination | Cell lines were tested negative for mycoplasma contamination. |
| Commonly misidentified lines<br>(See ICLAC register) | No commonly misidentified cell lines were used in the study. |

# Animals and other research organisms

Policy information about studies involving animals; ARRIVE guidelines recommended for reporting animal research, and Sex and Gender in Research

| | |
|---|---|
| Laboratory animals | Dnmt3a Villin-Cre mice (C57BL /6J background); male and females, aged 8-20 weeks were used in the study.<br>C57BL/6-Tg(CAG-Flpe)2Arte; male and females, age 8-10 weeks, were use for generation of Dnmt3a Villin-Cre mice.<br>Vil1-cre mice (C57BL/6J x SJL/J background); male, age 8-10 weeks were use for generation of Dnmt3a Villin-Cre mice. |
| Wild animals | The study did not involve wild animals. |
| Reporting on sex | For steady state investigations, we used male and females with an approx. ratio 1:1, distributed equally between WT and KO groups. For mouse experiments, we only used female mice, which is a common practice when investigating intestinal inflammation, and clearly indicated that in the methods section. |
| Field-collected samples | The study did not involve field-collected samples. |
| Ethics oversight | All animal studies were performed according to protocols approved by the Animal Investigation Committee of the University Hospital Schleswig-Holstein. |

Note that full information on the approval of the study protocol must also be provided in the manuscript.

# Flow Cytometry

## Plots

Confirm that:

☒ The axis labels state the marker and fluorochrome used (e.g. CD4-FITC).

☒ The axis scales are clearly visible. Include numbers along axes only for bottom left plot of group (a 'group' is an analysis of identical markers).

☐ All plots are contour plots with outliers or pseudocolor plots.

☒ A numerical value for number of cells or percentage (with statistics) is provided.

## Methodology

| | |
|---|---|
| Sample preparation | Cells were obtained from colonic lamina propria and mesenteric lymph nodes and stored on ice. Samples were reduced to single cell suspensions and resuspended in washing buffer and loaded into 96wells plated (V bottom) for further processing. After washing, Fc-blocking was performed and samples were surface stained using a cocktail of all the above described antibodies. A total of 50.000-200.000 cells were used for each sample measurement. For each sample, a negatively stained replicate was measured as negative control. |
| Instrument | SA3800 Spectral Analyzer (Sony Biotechnology) |
| Software | SA3800 Software version 2.0.5.54250. |
| Cell population abundance | 50.000-200.000 cells were loaded in each measured well. For each sample an unstained duplicate was included. Cell population abundances are intended as percentages of the previous gated population. |
| Gating strategy | Gating was performed excluding doublets and debris using FSC_H and FSC_A events distribution. Singlets were tested for viable population (Zombie Red negative) using using Zombie Red™ Fixable Viability Kit Biolegend 423109 (1:1000). Viable cells were plotted using CD45 as leukocyte marker. Positive CD45 events were tested for CD3 expression and CD3 positive events were plotted using CD4 and CD8 as T-cell markers for Cytotoxic and Helper T-cells. CD11b, CD11c, NK1.1 and CD19 were used as Monocytes, Dendritic cells, Natural Killer cells and B cells respectively. |

☒ Tick this box to confirm that a figure exemplifying the gating strategy is provided in the Supplementary Information.
